# Draft Genome Sequence of Staphylococcus epidermidis UMB8493, Isolated from the Female Urinary Tract

**DOI:** 10.1128/MRA.00419-20

**Published:** 2020-06-04

**Authors:** Anthony Truckenbrod, Taylor Miller-Ensminger, Adelina Voukadinova, Alan J. Wolfe, Catherine Putonti

**Affiliations:** a Department of Biology, Loyola University Chicago, Chicago, Illinois, USA; b Bioinformatics Program, Loyola University Chicago, Chicago, Illinois, USA; c Department of Microbiology and Immunology, Stritch School of Medicine, Loyola University Chicago, Maywood, Illinois, USA; d Department of Computer Science, Loyola University Chicago, Chicago, Illinois, USA; University of Maryland School of Medicine

## Abstract

Staphylococcus epidermidis is a native member of the human microbiota. Here, we report the draft genome sequence of S. epidermidis UMB8493, an isolate from a catheterized urine sample from a female with overactive bladder symptoms. The 2.54-Mbp draft genome encodes genes associated with beta-lactam and fosfomycin resistance.

## ANNOUNCEMENT

Staphylococcus epidermidis is a Gram-positive, coagulase-negative staphylococcus (CoNS) traditionally considered to be a benign commensal microorganism found on the skin and mucous membranes of humans and other mammals (1). More recently, however, S. epidermidis has been identified as being the leading causative agent of and most prominently recovered *Staphylococcus* species from nosocomial infections associated with indwelling medical devices, including pacemakers and catheters (1, 2). S. epidermidis has routinely been isolated from urine samples (3). Prior research confirmed that it is present in the bladder microbiota and is not a contaminant from the skin microbiota during collection (4). Its role in the urinary microbiota remains unknown; it has not been conclusively linked with lower urinary tract symptoms in adults, although it has been reported to cause urinary tract infections in children (5, 6). Here, we present the draft genome sequence of S. epidermidis UMB8493, isolated from a urine specimen collected via a transurethral catheter from a woman with overactive bladder (OAB) symptoms.

S. epidermidis UMB8493 was isolated from a prior institutional review board (IRB)-approved study (Loyola University Chicago, approval number LU207102) using the expanded quantitative urinary culture (EQUC) method (3). This sample was collected from a patient at Loyola University Medical Center’s Female Pelvic Medicine and Reconstructive Surgery Center (Maywood, IL, USA) in July 2018. The genus and species were determined using matrix-assisted laser desorption ionization–time of flight (MALDI-TOF) mass spectrometry following the previously described protocol (3) prior to storage at −80°C. From this freezer stock, the S. epidermidis isolate was streaked onto a Columbia nalidixic acid (CNA) agar plate and incubated at 35°C with 5% CO_2_ for 24 h. A single colony was selected and inoculated in Trypticase soy broth and incubated at 37°C for 24 h. DNA was extracted using the DNeasy blood and tissue kit following the manufacturer’s protocol for Gram-positive bacteria with the following exceptions: we used 230 μl of lysis buffer (180 μl of 20 mM Tris-Cl, 2 mM sodium EDTA, and 1.2% Triton X-100 and 50 μl of lysozyme) in step 2 and altered the incubation time in step 5 to 10 min. Upon purification, the genomic DNA was quantified using a Qubit fluorometer. DNA was then sent to the Microbial Genomic Sequencing Center (MiGS) at the University of Pittsburgh for sequencing. The libraries were prepared as follows: the DNA was first enzymatically fragmented using an Illumina tagmentation enzyme, and then indices were attached using PCR. The DNA library was sequenced using an Illumina NextSeq 550 platform, producing 2,584,013 pairs of 150-bp reads. Raw reads were trimmed using Sickle v1.33 (https://github.com/najoshi/sickle) and assembled using SPAdes v3.13.0 with the “only-assembler” option for K values of 55, 77, 99, and 127 (7). Genome coverage was calculated using BBMap v38.47 (https://sourceforge.net/projects/bbmap/). The NCBI Prokaryotic Genome Annotation Pipeline (PGAP) v4.11 (8) and PATRIC (9) were used to annotate the genome sequence. Unless previously noted, default parameters were used for each software tool.

The S. epidermidis UMB8493 draft genome sequence is 2,535,022 bp long assembled into 61 contigs with a GC content of 32%, genome coverage of 267×, and *N*_50_ score of 88,403 bp. The PGAP annotation identified 2,316 protein-coding genes, 10 rRNAs (4 5S, 4 16S, and 2 23S), and 58 tRNAs. PATRIC detected several genes associated with antibiotic resistance, including *blaZ* (beta-lactam resistance) and *fosB* (fosfomycin resistance). Future analyses of S. epidermidis will further our understanding of the epidemiology and transmission of this important commensal microorganism in the female urinary microbiome, allowing us to develop more effective therapeutic interventions in the future.

### Data availability.

This whole-genome shotgun (WGS) project has been deposited in GenBank under the accession number JAAUVT000000000. The version described in this paper is the first version, JAAUVT010000000. The raw sequencing reads have been deposited in the SRA under the accession number SRR11441022.
